# Alternatives to common approaches for training change of direction performance: a scoping review

**DOI:** 10.1186/s13102-022-00544-9

**Published:** 2022-08-03

**Authors:** Robert Buhmann, Max Stuelcken, Mark Sayers

**Affiliations:** School of Health and Behaviour Sciences, University of the Sunshine Coast, Maroochydore, QLD 4556 Australia

**Keywords:** Change of direction, Agility, Strength, Power, Kinematics

## Abstract

**Background:**

Research focuses heavily upon the effect of strength and power training on change of direction performance. The objective of this scoping review is to highlight alternative approaches to training change of direction.

**Methods:**

Four databases (Scopus, PubMed, Web of Science and SPORTDiscus) were searched with no date restrictions. To be included studies must (i) investigate change of direction performance following an intervention or investigate the relationships between variables of interest and change of direction performance; (ii) recruit participants > 18 years old; (iii) recruit participants involved in competitive sport. The majority of included studies investigated the effect of strength and/or power training, or, relationships between strength and/or power variables with change of direction performance.

**Results:**

Despite fewer studies, alternative training methods resulted in greater improvements (compared with strength and/or power) in change of direction performance, with smaller training durations. Few studies included reactive agility as an outcome measure.

**Conclusion:**

Despite much of the literature focusing on strength and/or power, there are alternative training modalities that demonstrate merit for improving change of direction performance. Future studies should investigate the effect of alternative training interventions on reactive agility performance, to provide a more valid indication of transfer to competition.

**Supplementary Information:**

The online version contains supplementary material available at 10.1186/s13102-022-00544-9.

## Introduction

The ability to change direction quickly is important for performance in many team and individual sports. Elite level players demonstrate faster change of direction times compared with sub-elite counterparts [27, 63] and a faster change of direction time may inform representative selection in younger age groups [28]. Additionally, recent work has highlighted that certain kinematic variables (e.g. trunk rotation and lateral flexion in the direction of the intended direction change) are mutually beneficial for faster change of direction times and reducing anterior cruciate ligament (ACL) injury risk [26]. A typical change of direction involves an athlete braking as quickly as possible when sprinting, followed by an acceleration in a different direction. These actions are pre-planned, or, in response to an external stimulus, which is termed reactive agility [11]. Given its association with higher levels of achievement in team sport and reduced injury risk [26], there is a strong focus in both research and applied settings on optimal methods for improving change of direction performance. Recent reviews cite the beneficial effect of resistance training [12, 13] on change of direction performance. However, some elite level clubs are reticent to adopt resistance training programs, particularly in periods of fixture congestion, possibly due to potential increases in player soreness and muscle damage [6]. Therefore, the programming of resistance training sessions is difficult during periods of fixture congestion, where teams often play two games per week. Resistance training programs also require relatively long periods of time (> 6 weeks) to elicit the desired muscular adaptations, making them difficult to incorporate into program structure once fixtures commence. Alternative training modalities for improving change of direction ability may have performance and injury prevention benefits. However, in comparison to strength and power training interventions, evidence supporting alternative methods of training change of direction performance is sparse.

Many studies investigating change of direction performance employ a pre-planned task as a primary outcome measure. It has been argued that reactive agility is a more valid measure of on field performance in comparison with pre-planned change of direction ability [68]. An understanding of factors influencing reactive agility performance, as opposed to pre-planned change of direction, may be of greater benefit to performance staff. Increasing strength and power has a positive impact on pre-planned change of direction [12, 13], however these factors are unlikely to have the same effect on reactive agility [68].

It should be acknowledged that there are capacities other than strength and/or power that can influence change of direction ability [19, 53]. For example, interventions aimed at training perception and decision making [53] and technique [19] report improvements in change of direction performance that exceed those reported in many studies involving strength or power focused interventions. The duration of these interventions [19, 53] is also short (three and six weeks respectively) in comparison with many strength and power training programs, which can last twelve weeks or longer [1, 4, 9, 25, 31, 65]; and the activities involved in these training interventions are less likely to result in soreness and high levels of muscle damage in trained athletes. There is an abundance of work investigating the use of strength training for improving change of direction performance. However, the impact of factors aside from strength and power on change of direction performance has not been reviewed. Understanding the factors aside from strength or power training influencing change of direction performance would benefit coaches, physical therapists and performance staff and help inform future research.

Despite the abundance of research on change of direction and reactive agility performance, the impact of factors aside from strength and power on change of direction performance has not been reviewed. The aim of this scoping review is to explore the literature for factors aside from strength and power that have an influence on pre-planned change of direction and reactive agility performance. A secondary aim is to recommend future avenues for research targeting an improvement in change of direction and reactive agility performance. The potential findings of this review may be useful for performance and sports medicine staff working with athletes who are required to change direction quickly by highlighting training methods or factors that are easy to implement in-season.

## Methods

### Search strategy

The systematic search was carried out on December 18th 2020, on Scopus, PubMed, Web of Science and SPORTDiscus databases with no date limit. Searches were limited to English and peer reviewed journal articles. The search strategies for all databases can be viewed in online (Additional file 1). The reference lists of recent systematic reviews were also searched to identify any articles missed by the original search. After the initial search, the lead investigator received automatically generated emails providing updates of results for the original search. These were received weekly with studies eligible for inclusion until December 17th 2021. Following searches, references were exported to EndNote^©^ (Version 9, Thomson-Reuters, Toronto, CA, USA).

### Inclusion criteria

The research question and inclusion criteria for the review were established using the PICOS (population, intervention, comparison, outcome and study design) model [16]. Only studies that investigated the effect of a targeted intervention on change of direction performance (pre-planned and reactive) in adult competitive athletes were included in this review. The outcome of interest was performance during a change of direction task (pre-planned or reactive). Studies examining relationships between change of direction performance and measures of strength, power, kinetics and/or kinematics were also included. Studies were excluded if the intervention was described as “stability” or “core” training, participants were < 18 years, or if participants were only recreationally trained. Studies including a “stability” or “core” training intervention lacked consistency in the type and volume of exercise prescribed, making it difficult to summarize the overall effect of this type of exercise on change of direction performance. Studies involving participants under eighteen were also excluded, as physical maturity impacts change of direction performance [51], potentially influencing the outcome of interventions. Studies including recreationally trained individuals (i.e. not involved in competitive sport at any level) were excluded as the ability to change direction quickly is a skill requiring training for high levels of performance. Therefore, studies recruiting participants not involved in competition may identify factors for performance that are not relevant for competitive athletes.

### Data extraction

The title and abstract of articles retrieved in the database search were screened by two authors (RB and MCS) to determine articles that were relevant for review. Once articles were screened, relevant data were charted in an Excel spreadsheet (Microsoft Excel, Microsoft, Redmond, DC, USA) [5]. Data that were charted from studies included authors, year of publication, training intervention, intervention duration and frequency, outcome task, difference in change of direction performance post intervention and correlation coefficients for variables related to change of direction performance. We chose to conduct a scoping review, as opposed to systematic review with meta-analysis, because our primary aim was to identify alternative approaches to strength and power training for improving change of direction performance (which we believe is a gap in the current literature). The effects of strength and power training on change of direction performance are well documented (e.g. [12]), however, the value of alternative approaches in change of direction training has not been clearly identified. We felt a scoping review was the most appropriate approach for achieving this aim because identifying and analysing knowledge gaps and identifying key factors related to a concept are important objectives for a scoping review [43]. Given that a universal tool for assessing bias of studies included in a scoping review is not currently available, a risk of bias assessment for the included studies was not undertaken. This is consistent with many previous scoping reviews and does not adversely affect the findings [5].

To summarize results, the number of studies employing interventions, or, investigating relationships with change of direction performance (as a percentage of total included studies in brackets) was reported. To explore training modalities that may be important for change of direction performance, interventions were first grouped by type (e.g. strength/power, technique) and the percentage change in the outcome task following the intervention was extracted from the study to calculate the mean change. The average percentage change in performance of post intervention outcome measures was reported, with the range (i.e. the minimum and maximum values reported in included studies), where possible, in brackets. Reporting a range was not always possible as there were instances where interventions were unique and could not be grouped. To explore factors that may be associated with change of direction performance the Pearson correlation coefficient was extracted from the study and reported. All factors strongly associated with performance were reported. Factors moderately associated with performance and observed by more than one study are reported in text. Factors moderately associated with performance and reported by a single study are reported in tables. Correlation coefficients were interpreted as follows 0.00–0.09 = negligible; 0.10–0.39 = weak; 0.40–0.69 = moderate; 0.70–0.89 = strong and 0.90–1.00 = very strong.

## Results

**Fig. 1:**
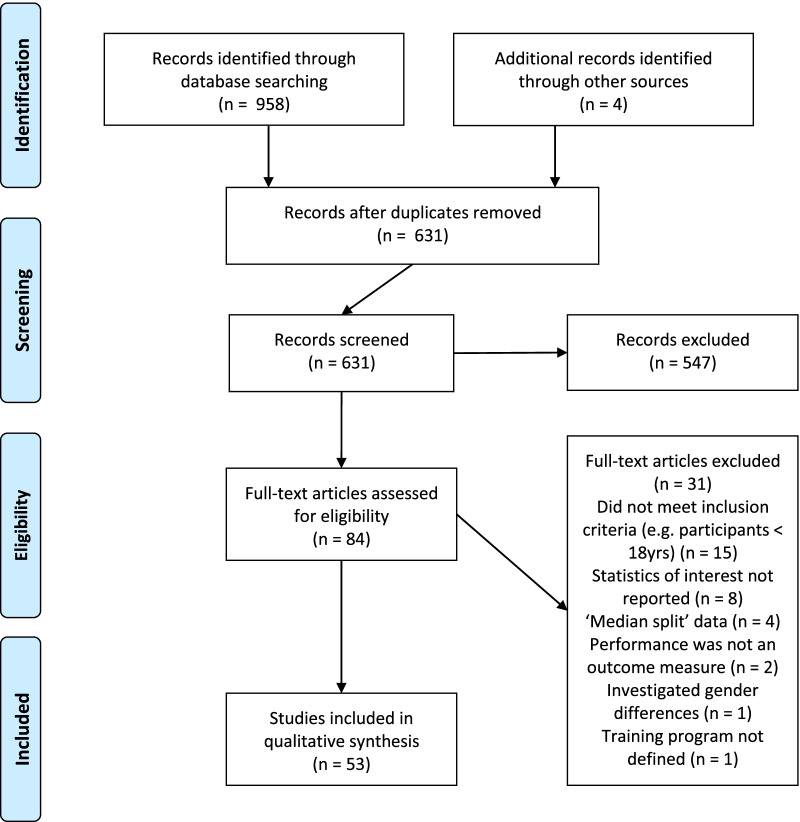
PRISMA diagram detailing the number of studies included/excluded in the review at each stage of the search

The study screening and inclusion process can be seen in Fig. 1. Fifty-three articles were selected for inclusion in this scoping review.

### Relationships between variables of muscle strength and power and change of direction performance

Seventeen studies (32% of included studies) investigated the relationship between strength and/or power and change of direction performance (Table 1). Variables sharing a strong relationship with faster pre-planned change of direction performance included broad jump distance (one study, r = − 0.80)[7], squat one repetition maximum relative to body weight (two studies, r = − 0.70 to − 0.85) [44, 60], countermovement jump height (one study, r = − 0.85) [46], force produced during an isometric mid-thigh pull (one study with multiple change of direction tests, r = − 0.79 to 0.85) [58], squat jump height (one study with multiple change of direction tests, r = − 0.70 to − 0.71) [61], countermovement jump height (one study, r = − 0.71) [61], absolute and relative hex-bar deadlift one repetition maximum (one study, r = − 0.72 and – 0.84 respectively) [62] and eccentric knee flexor strength (one study r = − 0.78) [29]. Variables sharing a moderate relationship with faster change of direction performance included those measured during a squat jump (force, height, power and velocity, four studies, r = − 0.38 to – 0.65) [46, 56, 60, 61], bilateral and unilateral countermovement jumps (height, force and power, three studies, r = − 0.48 to – 0.60) [23, 46, 61], drop jump (reactive strength, two studies, r = − 0.53 to – 0.65) [69, 71], isometric mid-thigh pull (force and rate of force development, one study, r = − 0.52 to – 0.66) [66] and vertical jump (power, one study, r = − 0.66) [7]. Lateral jump distance (two studies, r = − 0.42 to – 0.65) [17], eccentric knee flexor strength (one study, measured at different speeds, r = − 0.56 to – 0.64) [29] and concentric knee extension power (one study, r = − 0.54) [69] were also moderately related to faster pre-planned change of direction performance.

**Table 1:** Relationships between different variables and change of direction performance

Authors	N	Variables	COD task	Pre-planned/reactive	Relationships
Alemdaroglu 2012 [2]	12	Strength and power	T-test	Pre-planned	Force produced during a squat jump (r = – 0.47); force produced during a counter movement jump (r = – 0.59)
Banda et al. 2019 [7]	12	Power	Pro-agility test	Pre-planned	Average power produced during a vertical jump (r = 0.74), peak power produced during a vertical jump (r = 0.59), peak power produced during a vertical jump relative to body mass (r = – 0.66), broad jump distance (r = – 0.80)
Chaouachi et al. 2009 [14]	14	Power	T-test	Pre-planned	Total distance during a five horizontal jump test (r = – 0.61)
Delaney et al. 2015 [17]	31	Strength and power	505 test (changing direction to the dominant and non-dominant side)	Pre-planned	Squat one repetition maximum (kg) relative to body weight (r = – 0.52 for 505 dominant, r =– 0.55 for 505 non-dominant), lateral jump from dominant limb relative to height (r = – 0.34 for 505 dominant, r = – 0.65 for 505 non-dominant), lateral jump from non-dominant limb relative to height (r = – 0.42 for 505 dominant, r = – 0.56 for 505 non-dominant), peak power produced during a counter movement jump relative to body mass (r = – 0.47 for 505 dominant, r = – 0.48 for 505 non-dominant)
Dos'Santos et al. 2021 [19]	61	Kinetic and kinematic variables	cutting task with a 90° change of direction	Pre-planned	Greater velocity at final foot contact (r = – 0.75) and exit (r = – 0.73), faster approach velocity (r = – 0.66), greater peak (r = – 0.64) and mean (r = – 0.53) propulsive forces, greater medial lateral propulsive forces (r = – 0.58 to – 0.62), shorter approach time (r = 0.62), greater mean horizontal propulsive force (r = 0.60), shorter ground contact time at final and penultimate foot contact (r = 0.55 and 0.58) and greater mean horizontal braking forces at penultimate and final foot contact (r = 0.55 and 0.53)
Dos'Santos et al. 2020 [22]	61	Kinetic	505 test and modified 505 test (same as standard version except with a 20 m approach)	Pre-planned	Angle of resultant peak force (r = – 0.77), horizontal to vertical peak (r = 0.77) and mean (r = 0.74) propulsive ratio at final foot contact, horizontal to vertical peak (r = 0.51) and mean (r = 0.60) braking ratio at penultimate foot contact, peak hip flexion angle at penultimate foot contact (r = 0.59) and peak knee flexion angle at penultimate foot contact (r = – 0.51) associated with 505 test performance. Angle of resultant peak force (r = – 0.66), horizontal to vertical peak (r = 0.66) and mean (r = 0.68) propulsive ratio at final foot contact, horizontal to vertical peak (r = 0.51) and mean (r = 0.79) propulsive ratio at penultimate foot contact, angle of peak resultant braking force at final foot contact (r = – 0.57) and penultimate foot contact (r = – 0.55), peak (r = 0.59) and mean (r = 0.54) horizontal propulsive force at final foot contact all related to modified 505 performance
Dos'Santos et al. 2016 [20]	40	Kinetic	505 test and modified 505 test (same as standard version except with a 20 m approach)	Pre-planned	Vertical impact force at penultimate foot contact (r = 0.33), ground contact time at final foot contact (r = 0.75), vertical impact force at final foot contact (r = 0.55), horizontal braking force at final foot contact (r = 0.33) and horizontal propulsive force at final foot contact (r = – 0.61) related to modified 505 performance. Horizontal braking force at penultimate foot contact (r = – 0.33), vertical impact force at final foot contact (r = 0.44) and horizontal propulsive force at final foot contact (r = – 0.57) related to 505 test performance
Falch et al. 2020 [23]	23	Strength and power	Change of direction task at 45° or 180° with 4 m or 20 m approach	Pre-planned	Skate jump distance (r = – 0.49 for 45° COD with 4 m approach), height during a unilateral counter movement jump (r = – 0.60 for 180° change of direction with 4 m approach), distance during a skate jump (r = – 0.56 for 180° change of direction with a 4 m approach), reactive strength index during a drop jump (r = – 0.54 for 45° change of direction with 20 m approach), unilateral countermovement jump height (r = – 0.49 for 45° change of direction with 20 m approach), skate jump distance (r = – 0.76 and – 0.60 for 45° and 180° change of direction with 20 m approach)
Greig and Naylor. 2017 [29]	19	Strength	T-test and reactive cut	Pre-planned and reactive	Eccentric knee flexor strength at 60, 180 and 300°.s^−1^ (r = – 0.56, – 0.78 and – 0.64) related to pre-planned change of direction performance. Eccentric knee flexor strength at 60, 180 and 300°.s^−1^ not related to reactive agility performance (r = – 0.10 to – 0.14)
Havens and Sigward. 2015 [30]	25	Kinematic	Cutting tasks with direction changes at 45° and 90°	Pre-planned	Mediolateral centre of mass-centre of pressure separations (r = – 0.38), hip extensor moment (r = 0.39), hip sagittal power (r = – 0.47) and ankle plantar flexor moment (r = 0.45) associated with cutting performance at 45°. Mediolateral ground reaction force impulse (r = – 0.48), hip rotation angle (r = – 0.47), hip frontal power (r = – 0.58) and knee extensor moment (r = 0.49) associated with cutting performance at 90°
Jones et al. 2019 [41]	19	Kinetic	75° Cutting task	Pre-planned	Eccentric knee extensor moment (r = – 0.75) and eccentric knee flexor moment (r = – 0.54)
Marshall et al. 2014 [40]	15	Kinematic	Cutting task with a 75° direction change	Pre-planned	Maximum ankle power (r = 0.77), maximum ankle plantar flexor moment (r = 0.65), pelvis lateral tilt range (r = – 0.54), maximum lateral thorax rotation angle (r = 0.51), ground contact time (r = – 0.48)
McBurnie et al. 2019 [41]	34	Kinetic	Cutting task with change of direction at approximately 80°	Pre-planned	Peak knee rotation moment (r = 0.52) and peak knee flexion moment (r = – 0.51)
Nimphius et al. 2010 [44]	10	Strength and power	505 test (changing direction to the dominant and non-dominant side)	Pre-planned	Squat one repetition maximum relative to body weight (r = – 0.75, – 0.73 and – 0.85 for 505 non-dominant at pre-, mid- and post-season; r = 0.50, – 0.75 and – 0.60 for 505 dominant at pre-, mid- and post-season)
Pereira et al. 2018 [46]	38	Power	Zig-zag test and t-test	Pre-planned	Squat jump height (r = – 0.38, – 0.65) countermovement jump height (r = – 0.55, – 0.85) and mean propulsive power during a jump squat (r = – 0.40, – 0.50) related to zig zag test and t-test performance respectively
Santoro et al. 2021 [49]	40	Kinetic	505 test	Pre-planned	Linear regression model containing approach velocity, braking horizontal ground reaction force at final foot contact, braking vertical ground reaction force at final foot contact, propulsive horizontal ground reaction force at first accelerating foot contact, ground contact time at final foot contact, and propulsive vertical ground reaction force at first accelerating foot contact had an r value of 0.87
Sasaki et al. 2011 [50]	12	Kinematic	180° change of direction task	Pre-planned	Forward angular displacement of the trunk between foot contact and maximum trunk inclination (r = 0.61), ground contact time between foot contact and maximum trunk inclination (r = 0.65)
Scanlen et al. 2021 [52]	24	Strength and power	T-test	Pre-planned	Relative peak force during an isometric mid-thigh pull (r = 0.55), relative peak force during a countermovement jump (r = 0.62), standing long jump distance (r = 0.67)
Soslu et al. 2016 [56]	23	Strength	T-test	Pre-planned	Absolute force produced during a counter movement jump (r = – 0.58), absolute force produced during a squat jump (r = – 0.47)
Spiteri et al. 2015 [58]	12	Strength	T-test, 505 test and reactive basketball agility test	Pre-planned and reactive	Squat one repetition maximum (r = – 0.80 and – 0.80), maximal eccentric squat (r = – 0.87 and – 0.89), maximal concentric squat (r = – 0.79 and – 0.79) force produced during an isometric mid-thigh pull (r = – 0.85 and – 0.79) all related to t-test and 505 test performance respectively. Squat one repetition maximum (r = – 0.36), eccentric squat one repetition maximum (r = – 0.27), concentric squat one repetition maximum (r = – 0.27), isometric squat strength (r = – 0.08) and power produced during a countermovement jump (r = – 0.19) not significantly related to reactive agility performance
Swinton et al. 2014 [60]	30	Power	505 test	Pre-planned	Vertical jump height (r = – 0.54), squat one repetition maximum relative to body weight (r = – 0.70), average velocity during a squat jump (r = – 0.51), peak velocity during a squat jump (r = – 0.63), average power relative to body mass during a squat jump (r = – 0.40), peak power relative to body mass during a squat jump (r = – 0.45)
Thomas et al. 2017 [61]	26	Strength and power	505 test performed to the left and right	Pre-planned	Peak force during isometric mid-thigh pull (r = – 0.66 with 505 to the right), squat jump height (r = – 0.71 and – 0.70 with 505 to the left and right, countermovement jump height (r = – 0.71 and – 0.60 with 505 to the left and right)
Tramel et al. 2019 [62]	10	Strength	505 test and T-test	Pre-planned	Absolute (r = – 0.72) and relative (r = – 0.84) estimated hex-bar deadlift one repetition maximum related to t-test performance. Estimated hex-bar deadlift one repetition maximum relative to bodyweight related to 505 test performance to the right (r = – 0.68) and right (r = – 0.74)
Wang et al. 2016 [66]	15	Strength	Pro-agility test and T-test	Pre-planned	Peak rate of force development during isometric mid-thigh pull (r = – 0.52 with pro-agility), rate of force development at 30, 50, 90 and 100 ms (r = – 0.51, – 0.52, – 0.52 and – 0.51 all with pro-agility)
Welch et al. 2021 [67]	25	Kinematic	Cutting tasks with a 45° or 110° direction change	Pre-planned	For 45° cut, principal component (r = 0.26) interpreted as maintaining a low centre of mass during the concentric phase, shorter ground contact time, resisting reduction in lateral centre of mass to ankle and knee distance in the eccentric phase and using a faster and large extension of hip and knee. For 110° cut, first principal component (r = 0.66) was related to maintaining a low centre of mass during the concentric phase, using a shorter ground contact time, resisting a reduction in lateral centre of mass to ankle and knee distance in the eccentric phase, and resisting hip flexion the using hip extension. The second principal component (r = 0.27) was related to leaning in the direction of the cut
Young and Murray. 2017 [71]	19	Reactive strength	Offensive and defensive Reactive agility test for Australian Football	Reactive	Reactive strength measured during a drop jump (r = – 0.62 with defensive reactive agility, r = – 0.73 with offensive reactive agility)
Young. 2002 [69]	15	Reactive strength and power	Cutting tasks with direction changes at 20, 40 or 60°. Slalom with 4 × 60° cuts	Pre-planned	Reactive strength measured during a drop jump (r = – 0.65, – 0.53 and – 0.54 with cutting at 20 and 40°, and slalom performance). Concentric knee extension power (r = 0.54 with cutting at 40°)

Negative r values represent an improvement in change of direction completion time (unless otherwise stated)

Three studies (6% of included studies) investigated the association between markers of strength or power and reactive agility performance. One study measured eccentric knee flexor torque at several speeds on an isokinetic dynamometer and reported weak associations with reactive agility performance (r = – 0.10 to – 0.14) [29]. One study reported weak associations between measures of strength recorded during a back squat and reactive agility (r = – 0.08 to – 0.36) [58]. Another reported moderate and strong associations between the reactive strength index measured during a drop jump and reactive agility during a defensive and attacking Australian Rules Football drill (r = – 0.62 and – 0.73 respectively) [71].

### Effects of strength and power training interventions on change of direction performance

Twenty-four studies (45% of included studies) investigated the impact of a strength or power training intervention on change of direction performance (Table 2). Studies employing a strength or power training intervention reported an average change of – 3.4% (range = – 12 to 0.77%) in change of direction performance (a negative percentage change represents a reduction in change of direction task completion time, corresponding to an improvement in performance). All strength and/or power training studies employed a pre-planned change of direction task as the outcome measure. Six training studies (23% of all training studies) reported a significant group by time interaction [3, 15, 25, 32, 42, 55], indicating that strength or power training significantly improved change of direction performance over time compared with a control group. Six strength and/or power training studies (23% of all training studies) reported a significant effect of time and no differences between training and control groups following the intervention [24, 39, 45, 48, 57, 65]. Six strength and/or power training studies (23% of all training studies) reported no significant effect of a strength or power training intervention on change of direction performance [9, 33, 35, 36, 38, 54]. The back squat or leg press were the exercises most frequently used to train change of direction performance, being used in seventeen training studies (65% of all training studies) [1, 4, 8–10, 15, 24, 32, 35–37, 45, 48, 54, 57, 65]. Plyometrics/jumping exercises were the next most frequently used, being used in twelve studies (46% of all training studies) [23–25, 33, 36, 38, 39, 42, 48, 54, 64, 65]. The average duration of strength and/or power interventions was 8.5 weeks.

**Table 2:** The impact of training interventions on change of direction performance

Study	N	Training intervention	Training duration	Exercises	COD Test	Pre– planned or reactive	Percentage change in COD performance (mean [95%CI])	Significance
Abade et al. 2019 [1]	20	Strength and power	12 weeks (2 × weekly)	Horizontal leg press, bench press, 90° back squat. 4*6 repetitions at 80%1RM on one day, 4*8repetitions at 30%1RM on the other training day	6*20 m sprint with two 90° direction changes during each sprint	Pre-planned	– 1.9% [– 5.9 to 2.1%]	"Unclear". Used magnitude-based inference
Aloui et al. 2018 [3]	30	Strength	8 weeks (2 × weekly)	Isolated knee extension and hip extension using resistance bands	T-test	Pre-planned	– 6.6%	Effect of time in training group (*p* < 0.001, Cohen's d = 2.39). Significant group × time interaction (*p* = 0.007, Cohen's d = 0.12). Faster T-test time in strength group post intervention compared with controls
Appleby et al. 2020 [4]	33	Strength	18 weeks (2 × weekly)	Group 1: squat exercise; Group 2: step-up exercise	5 m sprint with a 50° direction change at 2.5 m	Pre-planned	Group 1: no change; Group 2: – 0.5%	"Unclear". Used magnitude-based inference
Banyard et al. 2021 [8]	24	Strength group and velocity based training group	6 weeks (3 × weekly)	Back squat (both groups)	505 test	Pre-planned	Strength group: – 3.5%; velocity group: – 5.2% (changes are average of tests performed turning to the dominant and non-dominant side)	"Possible" change in strength group. "Very likely" change in velocity group. Used magnitude-based inference
Barbalho et al. 2018 [9]	23	Strength	15 weeks (3 × weekly)	45° leg press, back squat, leg curl, calf raise, bench press, lat pull down, military press, seated cable row, triceps pully, biceps curl	T-test	Pre-planned	1.70%	No effect of time in training group (*p* = 0.58). No group × time interaction (*p* = 0.19)
Brito et al. 2014 [10]	57	Strength	9 weeks (2 × weekly)	Squat, calf raise, leg extension	T-test	Pre-planned	No change (values not reported)	No group × time interaction (*p* = 0.13, η^2^ = 0.10)
Coratella et al. 2019 [15]	40	Strength	8 weeks (1 × weekly)	Squat	T-test	Pre-planned	– 7% [– 12 to – 2%]	Significant group × time interaction (*p* = 0.01). Faster T-test time in strength group compared with control post intervention
Dos' Santos et al. 2021 [19]	15	Technique	6 weeks (2 × weekly)	Group 1: Technique training drills, program described in supplementary material of original article. Group 2: Sport and resistance training as normal	Change of direction tasks with 45° and 90° direction change	Pre-planned	Group 1: – 5.1%, group 2: no change (values not reported)	Effect of time in training group for cutting at 90° and 45° (*p* = 0.001 and 0.02). Significant group × time interaction for cutting at 90° and 45° (*p* < 0.001 and *p* = 0.003, η^2^ = 0.48 and 0.29). Times faster post intervention in training group compared with controls
Faude et al. 2013 [24]	16	Strength and power	7 weeks (2 × weekly)	Unilateral squats, hurdle jumps, bilateral squats, drop jumps, sprints, calf raises, high jumps, side lunge, lateral jumps, step ups, bounding	30 m sprint with 3 × 180° direction changes	Pre-planned	– 5%	Effect of time in training group (*p* = 0.005). No group × time interaction (*p* = 0.47, η^2^ = 0.04)
Fischetti et al. 2019 [25]	28	Power	12 weeks (3 × weekly)	Hurdle jumps, drop jumps, broad jumps	T-test	Pre-planned	– 2.6% [– 3.7 to 1.5%]	Effect of time in training group (*p* = 0.002). Significant group × time interaction (*p* < 0.001, Cohen's d = 0.73). T-test time faster in training group compared with control group post intervention
Hermassi et al. 2019 [32]	22	Strength and power	12 weeks (2 × weekly)	Snatch from a squat position, half-squat, clean and jerk	T-test	Pre-planned	– 11.6%	Effect of time (*p* = 0.002). Significant group × time interaction (*p* < 0.001, η^2^ = 0.58). Change of direction faster in training group compared with control post intervention
Hoffman et al. 2005 [33]	47	Power	5 Weeks (2 × weekly)	Group 1: only the concentric portion of the squat jump; group 2: concentric and eccentric potion of the squat jump	T-test	Pre-planned	Group 1: – 2.7%; group 2: – 1.8%	No effect of time, *p*-value not reported
Katushabe et al. 2020 [35]	17	Strength	8 weeks (2 × weekly)	Squat, Lunge, front squat, goblet squat, deadlift, sumo deadlift, nordice hamstring curls, single leg hip lifts	Zig-zag sprint course	Pre-planned	– 0.77%	No effect of time (*p* = 0.57). No group × time interaction)
Kobal et al. 2017 [36]	27	Strength and power	8 weeks (2 × weekly)	Half squat and drop jumps	505 test	Pre-planned	Changes not reported	No effect of time, *p*-value not reported
Kvorning et al. 2017 [37]	19	Strength and power	8 weeks (2 × weekly)	Power clean, squat, bench press, lat pull down, calf raise, leg curl, step up	T-test	Pre-planned	– 2.5%	T-test performance improved post intervention (*p* < 0.05). Study did not include control group
Lehnert et al. 2013 [38]	12	Power	6 weeks (3 × weekly)	Pogo jump, rim jump, side hop-sprint, lateral bound, knee-tuck jump, split jump, side hop, single leg lateral hop, scissors jump, diagonal bound, depth jump	T-test	Pre-planned	– 2.1%	No effect of time (*p* = 0.21). Study did not include a control group
Lockie et al. 2014 [39]	20	Power and deceleration	6 weeks (2 × weekly)	A-Skip, A-March, Partnered leg recovery, solo leg recovery, ankle hops, chute run, straight leg run, double hurdle run, run throughs split jump and flying 30 m sprint. Sprinting exercises invovled an enforced stopping distance	T-test	Pre-planned	– 4.4%	Effect of time (*p* = 0.03). No significant group × time interaction
Mohanta et al. 2019 [42]	40	Power	8 weeks (2 × weekly)	ankle hops, box jump, counter movement jump, hurdle jumps, lateral bounds, unilateral box jumps	T-test	Pre-planned	– 12.7%	Effect of time (*p* = 0.002). Significant group × time interaction (*p* < 0.001). T-test performance faster post intervention in training group compared with control group
O'Brien et al. 2020 [45]	20	Strength	4 weeks (2 × weekly)	Squat (group 1: no tempo restriction; group 2: 2 s concentric phase, 4 s eccentric phase)	505 test	Pre-planned	Group 1: – 3.7%; group 2: – 1.4%	Effect of time (*p* = 0.001). No group × time interaction
Raedergard et al. 2020 [48]	21	Strength and power	6 weeks (2 × weekly)	Group 1: squat, unilateral squat, calf raise, lateral lunge, unilateral nordic hamstring curl; group 2: drop jumps, unilateral counter movement jump, hurdle jumps, skate jumps, laying kick	COD from 45 to 180° with 4 m or 20 m approach	Pre-planned	Group 1: – 3.7 to 0.49% for all tests; group 2: – 4.6 to 0% for all tests	Pre to post intervention improvements observed in two of eight change of direction tests. No group by time interaction (*p* = 0.12, η^2^ = 0.13)
Serpell et al. 2011 [53]	15	Perception and decision making	3 weeks (2 × weekly)	Reactive agility drills	Rugby league specific test	Reactive and pre-planned	– 5.8% change in reactive agility performance. No change in pre-planned change of direction performance	Effect of time (*p* < 0.05). Significant group × time interaction (*p* < 0.05)
Shalfawi et al. 2013 [54]	20	Strength	10 weeks (2 × weekly)	Leg press, squat jump, nordic hamstring curl, leg extension, cable hip flexion, cable hip extension	S180° test	Pre-planned	– 0.5% [– 2.9 to 1.9%]	No effect of time, *p*-value not reported
Siddle et al. 2019 [55]	14	Strength	6 weeks (2 × weekly)	Nordic hamstring curl	20 m sprint with 180° direction change	Pre-planned	– 2.6% [– 5.7 to 0.5%]	Significant group × time interaction (*p* = 0.01, Cohen's d = 1.38). Improvement in change of direction performance was grater in training group compared with control group
Speirs et al. 2016 [57]	18	Strength	5 Weeks (2 × weekly)	Group 1: Bilateral squat; group 2: unilateral squat	Pro-agility test	Pre-planned	Group 1: – 1.7%; group 2: – 1.4%	Effect of time (*p* < 0.001). No significant group × time interaction
Vaczi et al. 2013 [64]	24	Power	6 weeks (2 × weekly)	Hurdle jumps, lateral jumps, single leg hop, depth jump, unilateral hurdle jump	T-test and Illinois agility test	Pre-planned	T-test: – 2.5%; Illinois agility test: – 1.7%	Group × time interaction (*p* < 0.05). Change of direction performance faster post intervention in training group compared with control group
Van den Tillaar et al. 2020 [65]	42	Strength and power	12 weeks (2 × weekly)	Squat jumps, calf hops, unilateral hops, unilateral squat jumps, squat, stand and flying start sprints (group 1: six weeks of squat and sprint training followed by six weeks of plyometric training, group 2: six weeks of plyometric training followed by six weeks of squat and sprint training	Handball specific change of direction test	Pre-planned	Group 1: 1% [– 8 to 10%]; group 2: 0% [– 10 to 10%]	Effect of time (*p* < 0.001). No significant group × time interaction

Negative values represent an improvement in change of direction time following intervention. Confidence intervals for the percentage change in performance are reported where available, confidence intervals for pre- versus post-intervention change are not reported in all studies. *p*-values are reported where available, where a *p*-value below a threshold was report a specific value could not be reported

### Relationships between kinetic and kinematic variables and change of direction performance

Nine (17% of included studies) investigated relationships between kinetic or kinematic variables and pre-planned change of direction performance [20–22, 34, 40, 41, 49, 50, 67] (Table 1). All kinetic or kinematic studies investigated relationships with pre-planned change of direction performance. Variables strongly associated with a faster change of direction time included greater centre of mass velocity at final foot contact (r = – 0.75) and exit from the direction change (r = – 0.73) [21], angle of resultant peak force (r = – 0.77) [22], mean (r = 0.77) and peak (r = 0.74) horizontal to vertical propulsive ground reaction force ratio at final foot contact [22], mean horizontal to vertical propulsive ground reaction force ratio at penultimate foot contact (r = 0.79) [22], shorter ground contact time at final foot contact (r = 0.75) [20], eccentric knee extensor moment (r = – 0.75) [34], and maximum ankle power (r = 0.77) [40]. Variables moderately associated with change of direction performance included mean and/or peak horizontal propulsive force (r = 0.54 to 0.61) [20–22], shorter ground contact time (r = 0.53 to 0.65) [21, 40, 50], ankle plantar flexor moment (r = 0.45 to 0.65) [30, 40] and knee flexor moment (r = – 0.54 to 0.51) [34, 41]. Several other variables were moderately related to change of direction performance, but only in one study (Table 1). One study used multiple linear regression to model change of direction performance [49]. The optimal model contained the variables approach velocity, horizontal braking ground reaction force at final foot contact, vertical ground reaction force during the braking phase of final foot contact, propulsive horizontal ground reaction force at first accelerating foot contact (the first step immediately following the direction change), ground contact time at final foot contact, and vertical ground reaction force during the propulsive phase of first accelerating foot contact (R^2^ = 0.75). One study used principle component analysis to identify important biomechanical components of change of direction performance [67]. This study identified a low centre of mass during the concentric phase, shorter ground contact time, resisting a reduction to lateral centre of mass to ankle and knee distance during the eccentric phase, and resisting hip flexion then using hip extension as important for cutting at 110°.

### Effects of alternative training interventions on change of direction performance

Two studies (8% of all training studies) used alternative training interventions to improve change of direction performance. One study used a technique training intervention with pre-planned change of direction as the outcome measure [19], the other study used a perceptual and decision making training intervention with reactive agility and pre-planned changed of direction as outcome measures [53]. These studies reported changes of – 5.1% [19] in pre-planned change of direction performance and – 5.8% [53] in reactive agility performance (Table 2). Both of these studies reported a group × time interaction [19, 53]; intervention duration was six [19] and three weeks [53] respectively.

## Discussion

The aim of this scoping review was to examine the literature for factors aside from strength and/or power training that improve change of direction performance. Most studies included in this review (81% of included studies) investigated relationships between strength and/or power variables and change of direction performance, or the effect of strength and/or power training on change of direction performance. Despite the emphasis on strength and/or power for improving change of direction performance, other training interventions may result in similar, or greater improvements. Studies targeting change of direction technique resulted in greater improvements in pre-planned change of direction performance (– 5.1%) [19] compared with the average improvement reported by studies employing strength and/or power interventions (– 3.4%). The other alternative intervention study, targeting perceptual and decision making skills, reported an improvement in reactive agility (– 5.8%) [53]; no other training studies used a reactive agility task as an outcome measure, making comparisons difficult. Given we have conducted a scoping review (not systematic review with meta-analysis) we are unable to determine which training method results in the best improvements in change of direction performance [5]. We are only able to highlight alternative methods (to traditional strength/power training) that may be more easily implemented in a team sport program. Additionally, studies investigating relationships between change of direction performance and factors other than strength and/or power (e.g. kinetics and kinematics) report strong relationships (r = – 0.73 to – 0.77; r = 0.74 to 0.79) [20–22, 34, 40] with several variables, similar to the strength of the relationships of some strength and/or power variables (r = – 0.85 to – 0.70; r = 0.85) [7, 29, 44, 46, 59–62]. Reactive agility was infrequently used as an outcome measure (7% of included studies); two studies reported weak relationships between reactive agility and strength/power variables (r = – 0.08 to 0.36) [29, 58] and one study reported moderate to strong relationships between the reactive strength index measured during a drop jump and reactive agility performance (r = – 0.62 to – 0.73) [71].

### Effects of alternative training approaches on change of direction performance

There were several variables, aside from those relating to strength and power that shared strong relationships with pre-planned changed of direction performance. Additionally, interventions targeting technique [19] and perceptual skills [53] were shorter in duration (six weeks [19] and three weeks [53]) than the average duration of strength and/or power interventions (approximately eight weeks). This suggests that there are training capacities, aside from strength and/or power, that can be trained in shorter time frames, resulting in similar effects on performance. The kinetic and kinematic variables strongly related to change of direction performance included a smaller angle of resultant peak force [22], increased velocity at specific points during a cutting manoeuvre [21], shorter ground contact time [20], greater eccentric knee extensor moment [34] and greater ankle power [40]. When performing a change of direction manoeuvre, these variables translate into an action that requires the performer getting lower to the ground (reducing the resultant angle of peak force), entering and exiting the manoeuvre at high speeds, taking short quick powerful steps and emphasizing plantarflexion. There are also several variables, related to large braking forces, that share a moderate correlation with change of direction performance [22]. These aspects of technique can be taught using drills within field-based training sessions, that are easier to program for performance staff (than resistance training programs, which require separate gym-based sessions), and potentially result in similar improvements in performance compared with strength and/or power training interventions alone. One study has successfully altered change of direction kinematics, resulting in improvements in performance, using a combination of external attentional focus and open/closed change of direction tasks [19]. These improvements occurred following a shorter training duration than typical strength and/or power training programs in the literature. Therefore, training programs aimed at improving change of direction technique are viable options for improving performance and may be more easily implemented than training programs that rely solely on weight room approaches to performance development.

### Practical recommendations

There are several suggestions that could improve change of direction performance in short training periods, based on studies investigating the effect of kinetic and kinematic variables with change of direction performance. There is merit in incorporating strength and power training for athletes required to change direction quickly, as greater eccentric knee extensor moments [34] and maximum ankle power [40] are strongly related to performance. Greater eccentric knee extensor moment may improve performance by increasing velocity and reducing ground contact time [34]; increased ankle power may also improve performance through reductions in ground contact time [40]. Therefore, exercises increasing eccentric strength of the knee extensors and power of plantar flexors will likely benefit pre-planned change of direction performance. Regarding kinematic variables, there are several cues that could be incorporated during field-based change of direction activities that would encourage performers to adopt beneficial kinematics during a direction change. For example the cue *“brake early/slam on the brakes”* has been used in one training study [19] to encourage athletes to reduce momentum quickly, resulting in higher velocities and shorter ground contact times (which are related to improved performance) at final foot contact [20, 22]. Another cue that has been used in a training intervention is *“cushion and push the ground away”* [19]. The aim of this cue is to increase propulsive forces, consequently increasing velocity and performance during a direction change [20, 22]. Finally, athletes could be encouraged to *“stay low”* and *“lean towards the intended direction change”.* These cues would encourage a more horizontal angle of resultant peak force during a direction change, which is also related to improved performance [22].

### Relationships between variables of strength/power and reactive agility

It is important to examine the relationship between measures of strength and/or power and reactive agility, as this outcome measure is likely to provide a more valid measure of on-field performance [71]. While several strength and/or power measures share strong relationships with pre-planned change of direction performance, strong relationships are not shared with reactive agility. Knee flexor torque [29] and back squat strength [58] share weak relationships with reactive agility performance. Agility performance is underpinned by two main components, the change of direction speed, and perceptual/decision making processes [47]. Measures of strength and power are likely to reflect an athlete’s change of direction speed, however, they do not reflect an athlete’s decision making and perceptual skills. One study reports moderate relationships between reactive strength index (measured during a drop jump) and reactive agility [71]. The reactive strength index is calculated as the jump height divided by the contact time [71] meaning athlete’s need to quickly jump following landing to improve this measure. Therefore, given they need to react quickly to initiate the jump response following landing, this variable may better reflect the perceptual component of reactive agility than other strength and power variables. Combined, these data highlight the “skill” component of reactive agility, which again reinforces the needs for drills in this domain to be highly sports specific.

### Future directions

A secondary aim of this scoping review was to determine areas for future research aimed at improving change of direction performance. The literature suggests that alternative approaches to improving change of direction, such as altering kinetics and kinematics [18–20, 22] and training perceptual skills [53]. Therefore, future large scale studies should investigate the influence of these interventions on change of direction ability, particularly using a reactive agility task as an outcome measure. Indeed, there are limited studies assessing factors important for change of direction that include a reactive agility task as an outcome measure. As most studies use a pre-planned change of direction task, they may not provide a valid indication of on-field performance [68]. To better understand factors important for on-field performance, future studies should include a reactive agility task. It is important to understand if improvements in performance (regardless of whether the intervention addresses strength, power, technique, or other capacities) transfer to different change of direction manoeuvres. Therefore, future studies should include a transfer test following the intervention to determine if the intervention results in performance transfer to different manoeuvres. Improvements in several tasks suggest better learning and are more likely to result in meaningful changes in on-field performance. Some studies investigating factors important for change of direction performance use questionable practices for determining factors important for performance. For example, many studies use the ‘median split’ method to split cohorts in half based on completion time during a change of direction task. These studies then report mean differences between ‘fast’ and ‘slow’ for several variables of interest, suggesting variables exhibiting large differences (effect sizes) are highly important for performance. Such methods can result in incorrect conclusions regarding the importance of specific variables for change of direction performance. For example, when simulating data based on a study using the ‘median split’ method [70], effect sizes (Cohen’s d) for variables of interest were large (i.e. > 0.80) when comparing the mean difference between groups. When using correlation to determine the association between the same variables and change of direction performance, correlations were moderate (r = 0.30–0.60). Therefore, use of the median split method to make inferences regarding the importance of certain variables for change of direction performance can result in inaccurate conclusions. Future studies should avoid the use of this methodology. Additionally, future studies should aim to increase sample size. The average sample size of included training studies (n = 26) and studies investigating relationships (n = 25) may be underpowered to detect small to moderate effects. Small to moderate effect sizes may be important during competition at the professional level, therefore studies should be appropriately powered to detect these differences.

## Conclusion

In conclusion, the literature focuses on using strength and/or power training and relationships with these variables when explaining improvements in change of direction performance. Most studies use a pre-planned change of direction task to evaluate responses to training, or when looking at relationships between variables. Despite limited studies, interventions employing alternative types of training (e.g. targeting kinematics) may have a similar, or greater effect on change of direction performance, with smaller program durations. Variables aside from those related to strength and/or power also share strong relationships with pre-planned change of direction performance. Training programs focusing on alternative approaches (to strength/power training) could be more easily implemented in congested schedules and their effect on change of direction performance should be explored in more depth. Future research should aim to investigate the effect of alternative methods of training on reactive agility performance, which may be a more valid indicator of on-field performance.

## Supplementary Information

**Additional file 1.** The search strategy used in each of the databases to identify articles for inclusion in the review.

## Data Availability

All data reported in this manuscript are from peer-reviewed publications. All of the extracted data are included in the manuscript.
