# Supplementary material for: Alternatives to common approaches for training change of direction performance: a scoping review
Source: BMC Sports Sci Med Rehabil. 2022 Aug 3;14:151. doi: 10.1186/s13102-022-00544-9 (PMC9347107; doi:10.1186/s13102-022-00544-9)
Supplement: Supplementary file 1 — Additional file 1. The search strategy used in each of the databases to identify articles for inclusion in the review. [file 13102_2022_544_MOESM1_ESM.pdf]

Appendix 1. Search strategies

Database: PubMed (MEDLINE)

Set #	Search terms	Results
1	(change of direction[ti] OR cutting[ti] OR agility[ti] OR reactive agility[tiab])	9,604
2	(biomech*[ti] OR kinematics[tiab] OR determinants[tiab] OR strength[ti] OR "rate of force development"[tiab] OR "resistance training"[tiab])	263,400
3	#1 AND #2	373
4	#3 Filters: Journal Article, English	323

Database: Scopus

Set #	Search terms	Results
1	(TITLE-ABS-KEY("change of direction" OR agility OR "reactive agility"))	19,277
2	(TITLE(biomech* OR kinematics OR strength))	275,558
3	#1 AND #2	338
4	#3 AND (LIMIT-TO(LANGUAGE , "English"))	325

Database: SPORTDiscus

Set #	Search terms	Results
1	TI "change of direction" OR AB "change of direction" OR TI agility OR AB agility OR TI "reactive agility" OR AB "reactive agility"	4,169
2	TI kinematics OR TI determinants OR TI strength OR TI "rate of force development" OR TI "resistance training"	32,263
3	#1 AND #2	327
4	#3 Narrow by language: English	297

Database: Web of Science:

Set #	Search terms	Results
1	TITLE: ("change of direction") OR TITLE: (agility) OR TITLE: ("reactive agility")	2,773
2	TITLE: (biomech*) OR TITLE: (kinematics) OR TITLE: (determinants) OR TITLE: (strength) OR TITLE: ("resistance training")	338,232
3	#1 AND #2	148
4	#2 AND #1 Refined by: LANGUAGES: (ENGLISH)	139
